# Double burden of malnutrition among children under five years in Saudi Arabia: a cross-sectional study of prevalence and determinants

**DOI:** 10.3389/fnut.2026.1830062

**Published:** 2026-05-29

**Authors:** Abdulaziz Fahad Alghofaili, Nesrin Kamal Abd El-Fatah

**Affiliations:** 1 Taif Postgraduate Training Program for Preventive Medicine, Ministry of Health, Taif, Saudi Arabia; 2 High Institute of Public Health, Alexandria University, Alexandria, Egypt

**Keywords:** obesity, preschool children, Saudi Arabia, stunting, underweight, wasting

## Abstract

**Background:**

Malnutrition among children under 5 years remains a major global public health concern. It encompasses both undernutrition and overnutrition, which can coexist within the same population. Estimating the prevalence of malnutrition among preschool children, will contribute to the limited body of city-level nutritional epidemiology studies in Saudi Arabia. This study aimed to assess the prevalence and associated factors of malnutrition among children under 5 years attending primary health care centers (PHCs) in Taif, Saudi Arabia.

**Methods:**

A cross-sectional study was conducted among 482 children under five attending selected PHCs in Taif, Saudi Arabia. Participants were selected using multistage random sampling. Anthropometric indicators including height-for-age, weight-for-height, weight-for-age, and BMI-for-age were calculated using WHO Child Growth Standards. Sociodemographic, perinatal, feeding, medical history, dietary habits, and lifestyle factors were assessed through caregiver interviews using a structured questionnaire. Logistic regression analysis was performed to identify associated factors of malnutrition.

**Results:**

The prevalence of stunting, wasting, underweight, overweight and obesity was 29.5%, 13.5%, 11.8%, 7.7%, and 11.6%, respectively, indicating the coexistence of undernutrition and overnutrition among preschool children. Several factors were found to be significantly associated with different forms of malnutrition. Low birth weight, early feeding before 6 months, recurrent infections, and <1 serving/day protein consumption were significantly associated with of stunting. Wasting was associated with lower protein consumption and increased screen time, while recurrent antibiotic use was associated with underweight. Overweight and obesity were associated with higher consumption of milk and dairy products, frequent fast-food intake, and recurrent diarrhea episodes.

**Conclusion:**

Multiple modifiable factors, including low birth weight, early feeding practices, recurrent childhood infections, dietary habits, and lifestyle behaviors, were associated with different forms of malnutrition. These findings underscore the need for targeted interventions focusing on improving infant feeding practices, dietary habits, lifestyle behaviors, and strengthening the prevention and management of recurrent childhood illnesses, with an emphasis on rational antibiotic use. Optimization and reinforcement of existing maternal and child health services, including antenatal care, routine growth monitoring, and caregiver-focused nutrition counseling at the primary health care level, may improve early detection and management of both undernutrition and overnutrition in early childhood.

## 1 Introduction

Early childhood malnutrition remains a major global health concern that includes both undernutrition (stunting, wasting, underweight) and overnutrition (overweight and obesity). Nutritional deficiencies during this stage can lead to impaired cognitive development, reduced educational attainment, and increased susceptibility to infectious and chronic diseases later in life. Globally, an estimated 150.2 million children under 5 years are stunted, 42.8 million are wasted, and 35.5 million are overweight in 2024, reflecting the persistent global burden of malnutrition (1).

The global prevalence of undernutrition reflects significant regional disparities. Meta-analytic evidence indicates that in low- and middle-income countries, stunting affects nearly 29.1% of under-five children, wasting about 6.3%, and underweight around 13.7%, with higher burdens in regions such as Western and Southern Asia and parts of Africa (2). In the broader Middle East and North Africa (MENA) region, an estimated 77 million children and adolescents experience some form of malnutrition, including both under- and overnutrition—a phenomenon referred to as the double burden of malnutrition, which denotes the coexistence of undernutrition (stunting, wasting, and underweight) and overnutrition (overweight/obesity) within the same population (3). Although Saudi Arabia has achieved substantial improvements in maternal and child health indicators, the persistence of stunting, wasting, micronutrient deficiencies, and emerging childhood overweight indicates that early childhood nutrition remains a public health priority (4).

In Saudi Arabia, the malnutrition profile among children under five reflects intermediate prevalence levels compared with global and regional estimates. In a large national sample, the prevalence of moderate and severe underweight was 6.9 and 1.3%, respectively; wasting was 9.8% and 2.9%, and stunting was 10.9% and 2.8% (4). Regional studies demonstrate substantial disparities within the country. In a representative cross-sectional analysis of <5 children from the Central, Southwestern (including Aseer), and Northern regions, prevalence of underweight was 4%, 19.7%, and 5.5%; wasting was 6.5%, 16.7%, and 6.5%; and stunting was 6.4%, 13.2%, and 6.4%, respectively, with significantly higher undernutrition in southwestern areas (5). Alongside undernutrition, an increasing prevalence of overweight and obesity has been observed among young children in Saudi Arabia. Although national preschool studies on overweight/obesity are more limited. Studies among kindergarten-aged children report overweight and obesity prevalence ranging from approximately 10 to 25% (6, 7). Longitudinal evidence also suggests declining severe undernutrition since the early 2000s, but a concurrent rise in overweight and obesity, reflecting the broader global nutrition transition (1). Comparative global data underscore that while Saudi Arabia has lower undernutrition prevalence relative to many low-income countries, its rates of stunting and wasting in preschool children remain higher than in high-income countries where wasting has fallen below 5% (5). Conversely, Saudi trends toward increasing overweight/obesity mirror those seen regionally in the Middle East/North Africa and globally, where overweight prevalence among children under 5 has risen significantly over the past decades (1).

Multiple determinants of childhood malnutrition have been identified, including socioeconomic factors, parental education, dietary practices, and environmental influences. Lower socioeconomic status and parental illiteracy are associated with higher prevalence of underweight and stunting, whereas higher socioeconomic status and urban residence correlate with increased overweight and obesity risk (8). Higher rates of childhood overweight have been reported in urban areas such as Riyadh and Jeddah, while southwestern regions like Aseer continue to show higher levels of undernutrition (3). Evidence from southwestern regions indicates that lower altitude and parental educational status are associated with higher prevalence of malnutrition among preschoolers (9). Dietary transitions characterized by increased consumption of energy-dense processed foods and reduced physical activity further contribute to further contribute to this trend (10). Micronutrient deficiencies, including iodine deficiency reported in the Aseer region, may further affect linear growth. Caregivers, particularly mothers, play a key role in shaping early childhood nutrition through feeding practices influenced by education, employment, and nutrition knowledge (11).

Despite multiple regional studies in Saudi Arabia, there is currently limited evidence specifically examining malnutrition among preschoolers in Taif city. Available Taif research to date has focused on older school-aged children (12, 13) and related nutritional issues, leaving a potential research gap in the assessment of early childhood malnutrition. Taif represents a unique setting due to its geographic characteristics, including higher altitude, as well as ongoing urbanization and lifestyle transitions that may influence dietary habits and physical activity. Variations in socioeconomic status may further contribute to differences in child nutritional outcomes. Studying preschool malnutrition in Saudi Arabia aligns with Saudi Vision 2030 and national health transformation goals that emphasize prevention, early intervention, and health equity. Therefore, this study aimed to estimate the prevalence and identify the associated factors of different forms of malnutrition (stunting, wasting, underweight, and overweight/obesity) among children under 5 years attending PHCs in Taif, Saudi Arabia. It was hypothesized that socioeconomic, dietary habits, physical activity, and health-related factors are significantly associated with nutritional status among this population.

## 2 Subjects and methods

### 2.1 Design and study population

A cross-sectional study was conducted among preschool children aged less than 5 years attending Well-Baby Clinics at Primary Health Care Centers (PHCs) in Taif city, between July 2025 and January 2026. The study population was intentionally restricted to children attending PHCs, as the objective was to assess malnutrition within this healthcare-utilizing population. Apparently healthy children whose caregivers consented to participate were included. Children with congenital anomalies, chronic diseases affecting growth, or physical deformities interfering with anthropometric measurements were excluded. In addition, Children with physical deformities interfering with anthropometric measurements were also excluded. Sample size was determined using EPI-INFO 7 software, a minimum required sample of 384 child aged less than 5 years was determined assuming that the prevalence of malnutrition among preschool children is 50% (there has been no prior estimate of the prevalence of malnutrition among in the Taif region) with a precision of 5% and confidence level of 95. After allowing an additional 25% to account for non-respondents, the final sample size was set at 482 children. Participants were selected using multistage random sampling methods. In Taif city, there are 19 PHC centers; four of them (nearly one-fourth) were selected randomly through the lottery method, taking into account logistical feasibility, resource constraints, and the need to ensure manageable and high-quality data collection within the study timeframe. The sampling frame included all eligible children attending the selected PHCs during the study period. Within each selected center, eligible children were recruited using systematic selection until the required sample size was achieved. The overall response rate was 94%. Ethical approval for the study was obtained from the Research Ethics Committee of Taif Health Affairs, Ministry of Health, Saudi Arabia (IRB. H-02-T-123, 2024-E-52). Written informed consent was obtained from parents or caregivers prior to participation. Confidentiality and anonymity of all participants were strictly maintained throughout the study.

### 2.2 Measures

Face-to-face interviews were conducted with parents or primary caregivers using a predesigned questionnaire developed based on prior literature on child malnutrition (14, 15) to identify sociodemographic characteristics, perinatal history, Child health-related factors, dietary habits, and physical activity patterns.

#### 2.2.1 Anthropometric measurements and nutritional status assessment

Anthropometric measurements were collected following standardized procedures recommended by the World Health Organization. Body weight was measured to the nearest 0.1 kg using a calibrated digital pediatric scale for children under 2-year-old and a digital weighing scale with the child wearing light clothing and no shoes. Standing height was measured to the nearest 0.1 cm using a portable infantometer for children under 2-year-old or stadiometer, with the child standing upright and the head positioned in the Frankfurt plane. Assessments were taken twice, and the average value was recorded. All measurements were performed by trained personnel following standardized procedures, and equipment was regularly calibrated to ensure accuracy.

Nutritional status was assessed using WHO Child Growth Standards (16). Height-for-age Z-scores (HAZ) were calculated to assess stunting, weight-for-age Z-scores (WAZ) to assess underweight, and weight-for-height Z-scores (WHZ) to assess wasting. Children with Z-scores below −3 standard deviations (SD) from the WHO reference median were classified as severely stunted, underweight, or wasted, while Z-scores between −3 and < −2 SD as moderately stunted, underweight, or wasted, respectively. Anthropometric indices were generated using WHO Anthro software (17). BMI-for-age Z-score (BAZ) was used to assess overweight and obesity in children under 5 years as recommended by WHO. As Weight-for-age does not differentiate between stunting and wasting and therefore is not suitable for evaluating excess weight. Children with BAZ > +2 SD were classified as overweight, while those with BAZ > +3 SD were classified as obese.

#### 2.2.2 Sociodemographic and early-life factors

Sociodemographic Information collected included the child’s sex, exact date of birth and birth weight. Family-related variables included monthly household income, parental educational level, parental occupation, number of household members, birth order of the child, place of residence (urban or rural), and marital status of parents. Maternal weight and height were also recorded to describe maternal nutritional status. Information regarding maternal and perinatal history was obtained from caregivers. Data collected included the mode of delivery, presence of pregnancy or delivery complications such as hypertension, gestational diabetes, or thyroid disorders, and the number of antenatal care visits during pregnancy. Infant feeding practices were assessed by collecting information on the type of feeding during the first 6 months of life, duration of breastfeeding and formula feeding, and age of introduction of complementary foods.

#### 2.2.3 Assessment of child health-related factors

Child health status was evaluated through caregiver-reported information on vaccination compliance, frequency of well-child clinic visits during the previous 2 years, and visits to health care providers during the last 6 months for minor illnesses. Information on recurrent antibiotic use was collected. Caregivers were also asked about the presence of acute or chronic health conditions, including recurrent infections, gastrointestinal symptoms, anemia, respiratory problems, dental issues, neurological or motor difficulties, and other chronic diseases.

#### 2.2.4 Assessment of dietary habits

Dietary habits were assessed using a structured caregiver-reported questionnaire. Information was collected on the regularity of breakfast intake, feeding difficulties such as poor appetite, overeating, or food selectivity, and whether the child followed a special diet or had food allergies. Data were also collected on pica behavior, feeding assistance, and eating with family. Use of iodized salt during cooking was assessed, along with the frequency of fast-food and soft drinks consumption, receipt of nutrition advice and dietary supplement use.

Food frequency intake of major food groups was assessed using a semi-quantitative food frequency framework commonly applied in epidemiological studies. Participants reported usual consumption of milk and dairy products, vegetables, fruits, protein sources, and cereals using standardized portion-based categories reflecting daily intake frequency (i.e., <1 serving/day, 1 serving/day, 2 servings/day, 3 servings/day, and ≥4 servings/day). Intake of discretionary foods, including fast foods and sugar-sweetened beverages, was assessed based on weekly consumption frequency and categorized as never, once per week, 2–3 times per week, and ≥4 times per week. These categorization schemes were adapted from validated dietary assessment approaches used in pediatric nutrition research. All response categories were standardized and clearly defined in the study questionnaire to minimize misclassification bias and improve reporting consistency. Although some variables may be conceptually related, they were treated as distinct constructs based on their theoretical relevance to child nutrition.

#### 2.2.5 Assessment of physical activity and screen time

Physical activity was assessed using a culturally adapted version of the Preschool-age Physical Activity Questionnaire (Pre-PAQ) (18). According to Guidelines of Beaton et al. (39), Forward translation was initially performed by two native Arabic language bilingual translators, who are fluent in English. A backward translation was then performed by two native English speaker translators, fluent in Arabic and unfamiliar with the concepts of the scales. The back-translated English questionnaire was subsequently compared with the original English one, then inconsistencies between the two versions were solved, to assure ensure linguistic accuracy. The questionnaire was reviewed and adapted to ensure suitability for the local context, including cultural appropriateness and relevance of activity examples. Caregivers were asked to report the average daily duration of the child’s engagement in various physical activities using predefined categorical time intervals (0, 15, 45, 90, and 150 min). For the purpose of quantitative analysis, these categories were converted into corresponding midpoint values to estimate total daily physical activity duration. This approach has been commonly used in similar epidemiological studies to facilitate statistical analysis.

Screen time was assessed using a caregiver-reported question on the child’s average daily time spent in screen-based activities, including television, smartphones, tablets, and computer games. Response options were categorized as: <30 min/day, 30–60 min/day, >1–2 h/day, and >2 h/day. Based on WHO guidelines, screen exposure is not recommended for children aged <2 years. For children aged 2–5 years, screen time was dichotomized into meeting recommendations (≤1 h/day) and not meeting recommendations (>1 h/day), with the latter classified as high screen time.

#### 2.2.6 Adoption and validation procedures

The questionnaire was developed based on previously validated instruments, including items adapted from Pre-PAQ, as well as established surveys addressing child nutrition, health history, and lifestyle behaviors. Face and content validity were ensured through expert review by specialists in pediatrics, community health, and nutrition, who evaluated the clarity, relevance, and cultural appropriateness of the items. Minor modifications were made based on their feedback. A pilot study was conducted on a sample of parents of preschool-aged children to assess clarity and feasibility. Internal consistency reliability of multi-item sections was evaluated using Cronbach’s alpha coefficient, which demonstrated good internal consistency (*α* = 0.827).

### 2.3 Statistical analysis

Data were entered, cleaned, and analyzed using IBM (SPSS) Statistics version 29.0 software. Prevalence of stunting, underweight, wasting, overweight, and obesity by age and sex was calculated according to WHO cut-off points. Descriptive statistics were used to summarize sociodemographic characteristics, dietary habits, and physical activity patterns. Continuous variables were presented as means and standard deviations after determining normality, while categorical variables were presented as frequencies and percentages. Associations between nutritional status indicators and sociodemographic variables, health and perinatal history, dietary habits, and physical activity levels were examined using chi-square tests for categorical variables and independent t-tests or one-way analysis of variance for continuous variables, as appropriate.

Multivariable logistic regression analysis was conducted to identify factors associated with stunting, wasting, underweight, and overweight/obesity. Independent variables showing a potential association in univariate analysis (*p* < 0.20) were considered for inclusion in the multivariable model. In addition, variables were selected *a priori* based on established determinants of child nutritional status reported in the literature to ensure adequate control for potential confounding. These included sociodemographic characteristics (child age, sex, parental education, occupation, household income, and family size), health-related factors, as well as dietary factors. All selected variables were entered simultaneously into the regression models to control for potential confounding effects. Adjusted odds ratios (AORs) with 95% confidence intervals (95% CIs) were calculated. Model fit was assessed using the Hosmer–Lemeshow goodness-of-fit test. Multicollinearity among independent variables was assessed using the variance inflation factor (VIF), with a cutoff value of <5 indicating no significant multicollinearity. Overall model significance was evaluated using the likelihood ratio chi-square test. A *p*-value <0.05 was considered statistically significant.

## 3 Results

**Figure 1:**
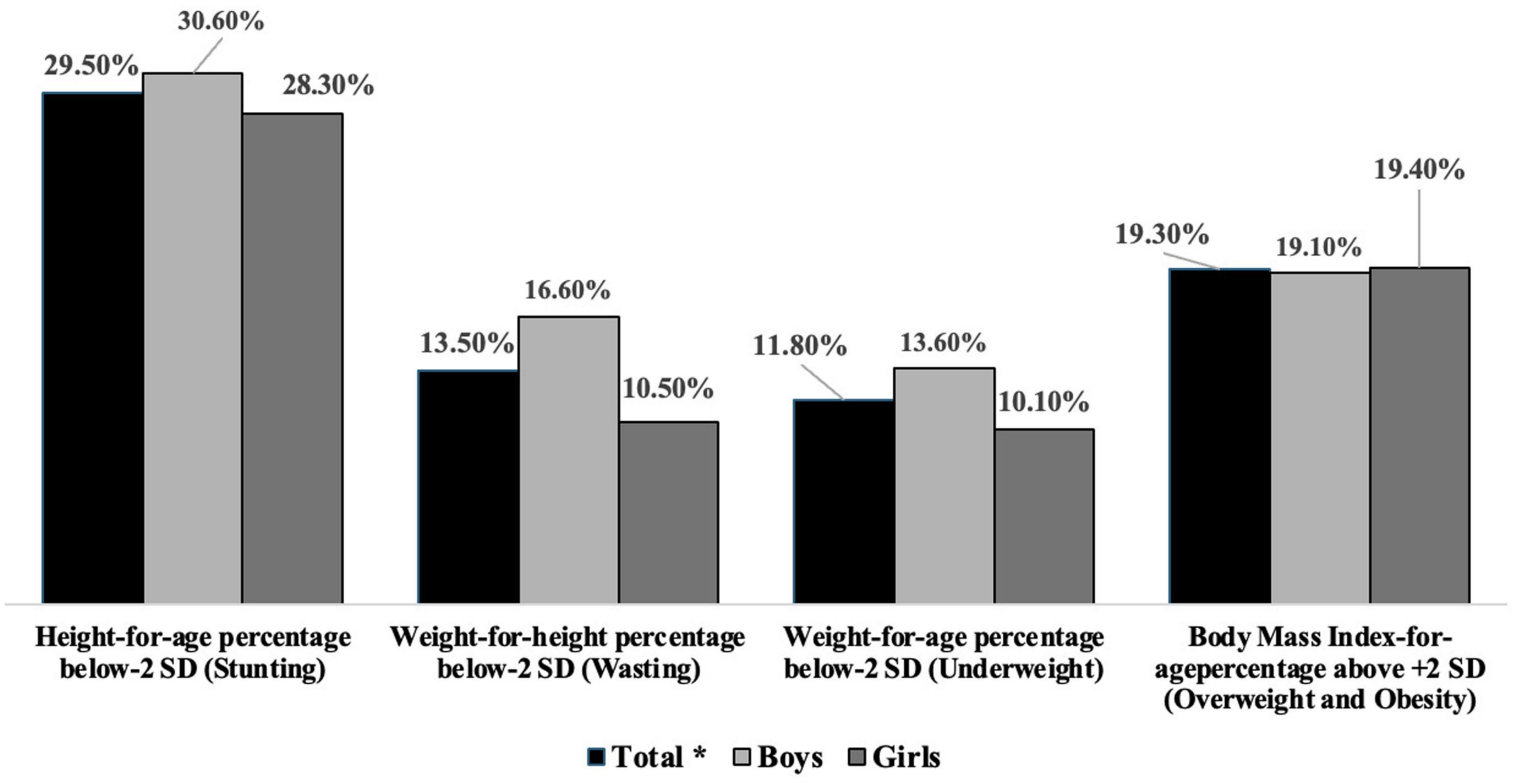
Percentage of children under five years of age whose nutritional status measures fell below −2 SD (height-for-age, weight-for-height, and weight-for-age) or above +2 SD (body mass index-for-age) according to the 2006 WHO Child Growth Standards reference median, Taif, 2026. *The total percentage includes children who are below −3 standard deviations (SD) from the WHO Child Growth Standards population median).

A total of 482 children under 5 years of age were included in this survey including 235 male (49%) and 247 female (51%). Nearly half of the participants (49.0%) aged from four to <5 years. Children aged 2–3 years represented 27.4% of the sample, while 21.2% were aged 3–4 years. The smallest proportion (2.5%) was observed among children aged 0–2 years. The anthropometric data analysis highlights the coexistence of undernutrition and overnutrition among under-five children in Taif, reflecting a double burden of malnutrition. Overall, 29.5% of the children were stunted, with 12.9% classified as moderately stunted and 16.6% as severely stunted. Wasting (weight-for-height < −2 SD) was identified in 13.5% of the children, including 7.1% who were moderately wasted and 6.4% who were severely wasted, indicating the presence of both chronic and acute forms of malnutrition. Underweight (weight-for-age < −2 SD) was detected in 11.8%, of whom 2.9% were severely underweight. Regarding BMI-for-age, 7.7% were overweight and 11.6% were obese reflecting a concurrent burden of overnutrition within the study population (Figure 1).

With respect to Sociodemographic data patterns in Table 1, moderate stunting peaked among children aged 0–<2 years, whereas severe stunting was proportionally higher among children aged 2–4 years. Children older than 4 years had lower rates of both moderate and severe stunting (8.9 and 14.0%, respectively). In contrast, wasting, underweight, and BMI-for-age did not show statistically significant variation among age groups. Girls had a lower prevalence of stunting, wasting and underweight; however, they showed a higher prevalence of overweight compared with boys. Family size, sex, birth order, residence, family income, parental education, parental occupation, and parental status were not significantly associated with all anthropometric indices.

**Table 1:** Percentage of children under 5 years classified as malnourished according to three anthropometric indices of nutritional status: height-for-age, weight-for-height, BMI-for-Age and weight-for-age, by sociodemographic characteristics, Taif 2026.

Sociodemographic Characteristics	Height for age		Weight-for-height		Weight-for-age		BMI-for-Age		Number of children (n = 482)	*p*-value
	Percentage below −2 SD^1^ (12.9%)	Percentage below −3 SD (16.6%)	Percentage below −2 SD^1^ (7.1%)	Percentage below −3 SD (6.4%)	Percentage below −2 SD^1^ (8.9%)	Percentage below −3 SD (2.9%)	Percentage between +2 and +3 SD (7.7%)	Percentage above +3 SD (11.6)		
Sex										
Male	13.6%	17.0%	8.5%	8.1%	9.4%	4.3%	6.4%	12.8%	235	P1 = 0.843 P2 = 0.145 P3 = 0.207 P4 = 0.183
Female	12.1%	16.2%	5.7%	4.9%	8.5%	1.6%	8.9%	10.5%	247	
Age in years										
0- < 2 years	33.3%	8.3%	8.3%	0.0%	0.0%	0.0%	16.7%	8.3%	12	P1 = 0.022* P2 = 0.603 P3 = 0.282^a^ P4 = 0.244
2–3 years	14.4%	18.9%	7.6%	6.8%	8.3%	3.8%	12.1%	9.8%	32	
3–4 years	17.6%	20.6%	7.8%	9.8%	14.7%	2.9%	4.9%	12.7%	102	
>4 years	8.9%	14.0%	6.4%	5.1%	7.2%	2.5%	5.9%	12.3%	236	
Family size										
≤Three	13.5%	15.9%	4.8%	7.1%	11.1%	0.8%	10.3%	8.7%	126	P1 = 0.777 P2 = 0.588 P3 = 0.200^a^ P4 = 0.713^a^
Four	14.0%	15.2%	8.4%	7.2%	9.6%	4.4%	7.2%	12.0%	250	
Six–seven	8.5%	22.0%	6.1%	2.4%	3.7%	2.4%	6.1%	14.6%	82	
≥Eight	12.5%	16.7%	8.3%	8.3%	8.3%	0.0%	4.2%	12.5%	24	
Birth order										
First child	12.8%	17.6%	6.4%	8.0%	8.8%	3.2%	8.8%	12.8%	125	P1 = 0.977 P2 = 0.946 P3 = 0.574^a^ P4 = 0.544
Second child	12.4%	14.0%	7.0%	4.7%	13.2%	3.1%	5.4%	7.8%	129	
Third child	13.9%	16.7%	7.4%	5.6%	5.6%	2.8%	5.6%	13.9%	108	
Fourd or more	12.5%	18.3%	7.5%	7.5%	7.5%	2.5%	10.8%	12.5%	120	
Residence										
Urban	12.7%	16.4%	7.3%	6.7%	9.1%	3.1%	7.3%	10.9%	450	P1 = 0.812 P2 = 0525^a^ P3 = 0.620^a^ P4 = 0.155^a^
Rural	15.6%	18.8%	3.1%	3.1%	6.3%	0.0%	12.5%	21.9%	32	
Household income										
≤5,000 SR	15.1%	16.4%	6.8%	4.1%	5.5%	4.1%	6.8%	6.8%	73	P1 = 0.855 P2 = 0.741 P3 = 0.693 P4 = 0.372
5,000–10.000 SR	14.2%	17.0%	5.7%	6.3%	8.5%	2.3%	9.7%	10.2%	176	
≥10.0 SR 00	11.2%	16.3%	8.2%	7.3%	10.3%	3.0%	6.4%	14.2%	233	
Father education										
Illiterate	13.3%	13.3%	6.7%	6.7%	6.7%	0.0%	6.7%	6.7%	15	P1 = 0.456^a^ P2 = 0.967^a^ P3 = 0.947^a^ P4 = 0.712^a^
Primary	33.3%	11.1%	0.0%	0.0%	0.0%	0.0%	0.0%	11.1%	9	
Secondary	11.5%	12.5%	6.7%	7.7%	9.6%	2.9%	12.5%	7.7%	104	
≥University	12.7%	18.1%	7.3%	6.2%	9.0%	3.1%	6.5%	13.0%	354	
Mother education										
Illiterate	25.0%	20.0%	10.0%	5.0%	10.0%	0.0%	15.0%	10.0%	20	P1 = 0.052^a^ P2 = 0.940^a^ P3 = 0.915^a^ P4 = 0.725^a^
Primary	0.0%	11.1%	0.0%	11.1%	0.0%	0.0%	0.0%	0.0%	9	
Secondary	10.4%	12.5%	6.3%	7.6%	8.3%	2.8%	9.7%	8.3%	144	
≥University	13.6%	18.4%	7.4%	5.8%	9.4%	3.2%	6.5%	13.6%	309	
Father occupation										
Unemployed	36.4%	9.1%	0.0%	9.1%	0.0%	9.1%	0.0%	0.0%	11	P1 = 0.3.44^a^ P2 = 0.819^a^ P3 = 0.319^a^ P4 = 0.212^a^
Retired	14.3%	7.1%	10.7%	3.6%	7.1%	3.6%	3.6%	7.1%	28	
Military	10.4%	15.6%	5.2%	5.9%	11.9%	0.0%	12.6%	8.1%	135	
Private work	14.3%	17.1%	10.0%	4.3%	8.6%	4.3%	7.1%	12.9%	70	
Government	12.6%	18.5%	7.1%	7.6%	8.0%	3.8%	5.9%	14.3%	238	
Mother occupation										
Housewife	10.9%	15.9%	8.9%	6.6%	7.9%	4.0%	8.6%	11.3%	306	P1 = 0.101 P2 = 0.179 P3 = 0.340^a^ P4 = 0.20^a^
Governmental	13.2%	19.1%	4.4%	7.4%	11.0%	1.5%	6.6%	13.2%	136	
Private work	25.0%	13.6%	2.3%	2.3%	9.1%	0.0%	4.5%	9.1%	44	
Parental status										
One or both parents died	12.5%	0.0%	0.0%	0.0%	0.0%	0.0%	12.5%	12.5%	8	P1 = 0.109^a^ P2 = 0.689^a^ P3 = 0.378^a^ P4 = 0.508^a^
Living together	12.1%	17.4%	7.4%	6.7%	9.6%	2.9%	8.0%	12.1%	448	
Separated	26.9%	7.7%	3.8%	3.8%	0.0%	3.8%	0.0%	3.8%	26	

Each of the indices is expressed in standard deviation units (SD) from the median of the WHO Child Growth Standards adopted in 2006. Table is based on children with valid dates of birth (month and year) and valid measurement of both height and weight. Bold values indicate statistical significance.

1 Includes children who are between −2 and −3 standard deviations (SD) from the WHO Child Growth standards population median.

a Monte-Carlo corrected *p*-value, *p-value < 0.05.

P1: Height-for-age; P2: Weight-for-height; P3: Weight-for-age; P4: BMI-for-age.

In Table 2, Low birth weight showed a significant association with stunting, wasting, and obesity. Children with a history of low birth weight demonstrated higher proportions of severe stunting and wasting. Early feeding practice before 6 months was significantly associated with stunting. Mixed feeding was associated with higher moderate stunting rates compared with exclusive breastfeeding, while severe stunting was highest among exclusively breastfed children (20.4%). Children receiving formula for less than 1 year of child age are more likely to be stunted. Age of introduction of solid foods was significantly related to BMI-for-age, with delayed introduction (after 12 months) associated with higher obesity percentages (16.8%). Recurrent antibiotic use was significantly associated with lower wasting and underweight rates. A history of recurrent diarrhea showed also significant associations with markedly higher severe stunting (35.3%) and obesity (23.5%) among affected children. Considering Dental caries, children with caries had higher severe stunting (42.4%) and severe underweight percentages. Psychological diseases were significantly associated with weight-for-age (P3 = 0.030), although the number of affected cases was small.

**Table 2:** Percentage of children under 5 years classified as malnourished according to three anthropometric indices of nutritional status: height-for-age, weight-for-height, BMI-for-Age and weight-for-age, by perinatal, Early Feeding and medical histories, Taif 2026.

Medical and pregnancy histories	Height for age		Weight-for-height		Weight-for-age		BMI-for-Age		Number of children (*n* = 482)	*p*-value
	Percentage below −2 SD^1^ (12.9%)	Percentage below −3 SD (16.6%)	Percentage below −2 SD (7.1%)	Percentage below −3 SD (6.4%)	Percentage below −2 SD (8.9%)	Percentage below −3 SD (2.9%)	Percentage between +2 and +3 SD (7.7%)	Percentage above +3 SD (11.6)		
Low birth weight										
No	10.8%	12.3%	4.9%	5.6%	8.0%	2.2%	7.7%	10.2%	324	**P1 = 0.000**^**a**^ **P2 = 0.014** P3 = 0.215 **P4 = 0.015**
Yes	17.1%	25.3%	11.4%	8.2%	10.8%	4.4%	7.6%	14.6%	158	
Delivery mode										
Cesarian	12.0%	16.5%	7.3%	6.7%	9.0%	3.4%	8.4%	12.9%	357	P1 = 0.844^a^ P2 = 0.954^a^ P3 = 0.783^a^ P4 = 0.474^a^
Normal	14.9%	16.5%	6.6%	5.8%	9.1%	1.7%	5.0%	8.3%	121	
Forceps	25.0%	25.0%	0.0%	0.0%	0.0%	0.0%	25.0%	0.0%	4	
Pregnancy complication										
No	12.8%	16.3%	7.4%	6.9%	9.4%	3.3%	6.9%	11.2%	392	P1 = 0.926 P2 = 0.552 P3 = 0.357 P4 = 0.604
Yes	13.3%	17.8%	5.6%	4.4%	6.7%	1.1%	11.1%	13.3%	90	
Mother’s nutritional status in terms of BMI										
Underweight	16.7%	16.7%	16.7%	0.0%	0.0%	0.0%	16.7%	16.7%	6	P1 = 0.539^a^ P2 = 0.670 P3 = 0.167^a^ P4 = 0.178^a^
Normal	14.1%	19.1%	7.5%	8.5%	12.6%	2.0%	5.5%	6.5%	199	
Overweight	10.1%	13.8%	6.9%	4.8%	4.8%	3.7%	8.0%	15.4%	188	
obese	15.7%	16.9%	5.6%	5.6%	10.1%	3.4%	11.2%	14.6%	89	
Mother antenatal care visits										
No visits	10.0%	10.0%	5.0%	0.0%	10.0%	5.0%	15.0%	15.0%	20	P1 = 0.752 P2 = 0.643 P3 = 0.950^a^ P4 = 0.306
1_2 visits	14.6%	13.5%	9.0%	3.4%	11.2%	3.4%	5.6%	6.7%	89	
3_4 visits	10.6%	16.2%	6.3%	7.0%	7.0%	2.8%	9.9%	12.0%	142	
>4visits	13.9%	18.6%	6.9%	7.8%	9.1%	2.6%	6.5%	13.0%	231	
Early feeding before 6 months										
Exclusive breast feeding	9.2%	20.4%	6.6%	5.3%	8.6%	2.6%	7.9%	7.9%	152	**P1 = 0.034** P2 = 0.506^a^ P3 = 0.241^a^ P4 = 0.479
Formula feeding	9.8%	6.6%	8.2%	11.5%	16.4%	1.6%	4.9%	13.1%	61	
Mixed feeding	15.6%	16.7%	7.1%	5.9%	7.4%	3.3%	8.2%	13.4%	269	
Breastfeeding duration										
No breastfeeding	9.8%	6.6%	8.2%	11.5%	16.4%	1.6%	4.9%	13.1%	61	P1 = 0.247 P2 = 0.888^a^ P3 = 0.516^a^ P4 = 0.911^a^
Less than 1 year	15.9%	16.3%	7.2%	6.3%	6.3%	3.4%	8.7%	12.0%	208	
1 year	11.5%	20.4%	7.1%	5.3%	9.7%	2.7%	8.8%	12.4%	113	
1.5 year	11.8%	17.6%	5.9%	4.4%	7.4%	2.9%	8.8%	8.8%	68	
2 years	6.3%	21.9%	6.3%	6.3%	12.5%	3.1%	0.0%	9.4%	32	
Formula feeding duration										
No formula feeding	9.2%	20.4%	6.6%	5.3%	8.6%	2.6%	7.9%	7.9%	152	**P1 = 0.049** P2 = 0.831^a^ P3 = 0.752^a^ P4 = 0.241^a^
Less than 1 year	18.5%	33.3%	3.7%	3.7%	7.4%	3.7%	3.7%	25.9%	27	
1 year	14.9%	14.9%	8.5%	2.1%	8.5%	6.4%	14.9%	6.4%	47	
1.5 year	12.6%	16.5%	7.1%	7.9%	11.8%	3.1%	7.1%	12.6%	127	
2 years	15.5%	9.3%	7.8%	8.5%	7.0%	1.6%	6.2%	14.0%	129	
Age of introduction of solid foods										
At 6 months	10.7%	16.8%	8.7%	8.2%	10.7%	2.0%	5.1%	14.3%	196	P1 = 0.508 P2 = 0.669 P3 = 0.233^a^ **P4 = 0.012**^**a**^
7–8 months	14.4%	13.3%	6.7%	4.4%	12.2%	4.4%	4.4%	2.2%	90	
9–10 months	18.2%	20.8%	3.9%	5.2%	7.8%	1.3%	11.7%	7.8%	77	
After 12 months	11.8%	16.0%	6.7%	5.9%	4.2%	4.2%	11.8%	16.8%	119	
Immunization status										
No	11.8%	11.8%	5.3%	10.5%	7.9%	1.3%	9.2%	9.2%	76	P1 = 0.421 P2 = 0.244 P3 = 0.619 P4 = 0.787
Yes	13.1%	17.5%	7.4%	5.7%	9.1%	3.2%	7.4%	12.1%	406	
Well baby clinic visits before 2 years										
No visit	21.3%	11.5%	6.6%	3.3%	4.9%	1.6%	11.5%	16.4%	61	P1 = 0.275 P2 = 0.816 P3 = 0.313^a^ P4 = 0.768
1–2visits	11.0%	15.2%	6.2%	7.6%	9.5%	1.4%	7.6%	11.4%	210	
3–4visits	11.5%	18.0%	7.4%	7.4%	9.0%	5.7%	7.4%	11.5%	122	
More than 4 visits	13.5%	21.3%	9.0%	4.5%	10.1%	3.4%	5.6%	9.0%	89	
Doctor visits in the previous 6 months										
No	9.6%	15.9%	8.9%	7.6%	7.6%	4.5%	4.5%	12.1%	157	P1 = 0.277 P2 = 0.381 P3 = 0.307 P4 = 0.165
Yes	14.5%	16.9%	6.2%	5.8%	9.5%	2.2%	9.2%	11.4%	325	
Recurrent antibiotic use										
No	12.9%	16.5%	8.2%	6.2%	10.3%	3.1%	7.2%	11.5%	418	P1 = 0.988 **P2 = 0.047**^**a**^ **P3 = 0.019** P4 = 0.222^a^
Yes	12.5%	17.2%	0.0%	7.7%	0.0%	1.6%	10.8%	12.3%	64	
History of recurrent infection/fever										
No	12.3%	15.6%	8.2%	6.9%	10.0%	3.1%	6.7%	10.8%	390	P1 = 0.320 **P2 = 0.**073 P3 = 0.198 P4 = 0.102
yes	15.2%	20.7%	2.2%	4.3%	4.3%	2.2%	12.0%	15.2%	92	
History of recurrent diarrhea										
No	13.2%	15.2%	7.6%	6.7%	9.6%	2.9%	7.1%	10.7%	448	**P1 = 0.010** P2 = 0.147^a^ P3 = 0.152^a^ **P4 = 0.038**^**a**^
Yes	8.8%	35.3%	0.0%	2.9%	0.0%	2.9%	14.7%	23.5%	34	
Anemia										
No	12.8%	16.6%	7.2%	6.3%	8.8%	2.7%	7.8%	11.8%	475	P1 = 1.000^a^ P2 = 0.432^a^ P3 = 0.207^a^ P4 = 0.591^a^
Yes	14.3%	14.3%	0.0%	14.3%	14.3%	14.3%	0.0%	0.0%	7	
Chronic disease										
No	13.0%	16.7%	7.1%	6.5%	9.0%	2.9%	7.7%	11.5%	478	P1 = 0.449^a^ P2 = 1.000^a^ P3 = 1.000^a^ P4 = 1.000^a^
Yes	0.0%	0.0%	0.0%	0.0%	0.0%	0.0%	0.0%	25.0%	4	
Dental caries										
No	12.9%	14.7%	6.9%	6.2%	8.7%	2.2%	8.0%	10.9%	449	**P1 = 0.000** P2 = 0.799^a^ **P3 = 0.009**^**a**^ P4 = 0.167^a^
Yes	12.1%	42.4%	9.1%	9.1%	12.1%	12.1%	3.0%	21.2%	33	
Psychological diseases										
No	12.9%	16.4%	7.1%	6.4%	8.9%	2.7%	7.7%	11.6%	481	P1 = 0.293^a^ P2 = 1.000^a^ **P3 = 0.030**^a^ P4 = 1.000^a^
Yes	0.0%	100.0%	0.0%	0.0%	0.0%	100.0%	0.0%	0.0%	1	
Neurological diseases										
No	13.0%	16.7%	7.1%	6.3%	9.0%	2.9%	7.7%	11.7%	478	P1 = 0.449^a^ P2 = 0.264^a^ P3 = 1.000^a^ P4 = 0.482^a^
yes	0.0%	0.0%	0.0%	25.0%	0.0%	0.0%	0.0%	0.0%	4	

Each of the indices is expressed in standard deviation units (SD) from the median of the WHO Child Growth Standards adopted in 2006. Table is based on children with valid dates of birth (month and year) and valid measurement of both height and weight. Bold values indicate statistical significance.

1 Includes children who are between −2 and −3 standard deviations (SD) from the WHO Child Growth standards population median.

a Monte-Carlo corrected *p*-value, *p-value < 0.05.

P1: Height-for-age; P2: Weight-for-height; P3: Weight-for-age; P4: BMI-for-age.

Table 3 shows that, children who required assistance during feeding showed higher rates of stunting and underweight and children who did not eat with their family had markedly higher wasting and underweight percentages. Feeding problems were significantly associated with stunting, particularly among picky eaters. Likewise, Children who received advice from a nutritionist showed lower stunting percentages. Regarding Dietary habits, Lower milk and yoghurt consumption was significantly associated with wasting and underweight, whereas higher intake (≥4 cups/day) was linked with increased obesity. Children consuming <1 serving per day protein foods had the highest stunting (27.8%) and wasting (22.2%) rates, and cereal consumption with irregular patterns linked to higher wasting. On the other hand, children consuming fast food more than three times per week had the highest obesity prevalence (45.0%) and significantly higher stunting rates. Pica practice showed a significant association with BMI-for-age, with higher obesity percentages among affected children. Finally irregular dietary supplement use was significantly related to weight-for-age, and timing of initiation was associated with weight-for-height.

**Table 3:** Percentage of children under 5 years classified as malnourished according to three anthropometric indices of nutritional status: height-for-age, weight-for-height, BMI-for-Age and weight-for-age, by children’s dietary habits, Taif 2026.

Dietary habits	Height for age		Weight-for-height		Weight-for-age		BMI-for-age		Number of children (*n* = 482)	*p*-value
	Percentage below −2 SD^1^ (12.9%)	Percentage below −3 SD (16.6%)	Percentage below −2 SD (7.1%)	Percentage below −3 SD (6.4%)	Percentage below −2 SD (8.9%)	Percentage below −3 SD (2.9%)	Percentage between +2 and +3 SD (7.7%)	Percentage above +3 SD (11.6)		
Child on a special diet										
No	12.7%	16.4%	7.1%	6.3%	8.9%	2.8%	7.6%	11.2%	463	P1 = 0.816^a^ P2 = 0.768^a^ P3 = 1.000^a^ P4 = 0.611^a^
Yes	15.8%	21.1%	5.3%	10.5%	10.5%	5.3%	10.5%	21.1%	19	
Food allergy history										
No	13.0%	16.9%	7.2%	6.5%	9.4%	2.7%	8.1%	11.5%	445	P1 = 0.775 P2 = 0.936^a^ P3 = 0.228^a^ P4 = 0.803^a^
Yes	10.8%	13.5%	5.4%	5.4%	2.7%	5.4%	2.7%	13.5%	37	
Level of feeding assistance										
Eats independently	10.5%	12.3%	7.3%	8.2%	9.6%	2.3%	5.5%	12.3%	219	**P1 = 0.046** **P2 = 0.039** **P3 = 0.018** P4 = 0.605
Eats with assistance	15.9%	23.2%	13.4%	4.9%	17.1%	3.7%	11.0%	8.5%	82	
Fully assisted	14.4%	18.8%	3.9%	5.0%	4.4%	3.3%	8.8%	12.2%	181	
Eating with family										
No	33.3%	33.3%	100.0%	0.0%	66.7%	33.3%	0.0%	0.0%	3	P1 = 0.452^a^ **P2 = 0.000**^**a**^ **P3 = 0.003**^**a**^ P4 = 0.710^a^
Sometimes	14.9%	18.8%	7.9%	5.0%	9.9%	4.0%	9.9%	15.8%	101	
Yes	12.2%	15.9%	6.1%	6.9%	8.2%	2.4%	7.1%	10.6%	378	
Number of daily meals										
Less than 3 meals	15.4%	17.3%	7.7%	7.7%	13.5%	5.8%	9.6%	11.5%	52	P1 = 0.325 P2 = 0.993^a^ P3 = 0.083^a^ P4 = 0.872
3 meals	10.9%	15.4%	7.1%	6.4%	10.0%	1.9%	7.4%	10.0%	311	
More than 3 meals	16.8%	19.3%	6.7%	5.9%	4.2%	4.2%	7.6%	16.0%	119	
Breakfast intake										
Does not	28.6%	14.3%	14.3%	0.0%	14.3%	0.0%	0.0%	14.3%	7	P1 = 0.862^a^ P2 = 0.246^a^ P3 = 0.761^a^ P4 = 0.652^a^
Rarely	18.5%	11.1%	18.5%	3.7%	3.7%	7.4%	14.8%	3.7%	27	
3–4 times	11.3%	18.3%	9.9%	7.0%	9.9%	4.2%	7.0%	8.5%	71	
5–6 times	10.9%	19.3%	7.6%	7.6%	10.1%	3.4%	8.4%	10.9%	119	
Daily	13.2%	15.5%	4.7%	6.2%	8.5%	1.9%	7.0%	13.6%	258	
Feeding problems										
No	11.9%	18.4%	6.8%	6.8%	9.2%	2.4%	7.1%	11.3%	337	**P1 = 0.029** P2 = 0.299^a^ P3 = 0.623^a^ P4 = 0.286^a^
Poor appetite	10.2%	22.4%	14.3%	4.1%	8.2%	6.1%	10.2%	14.3%	49	
Difficulty eating	0.0%	7.1%	0.0%	0.0%	0.0%	0.0%	14.3%	35.7%	14	
Picky eater	20.7%	7.3%	4.9%	7.3%	9.8%	3.7%	7.3%	7.3%	82	
Received any nutrition advice										
No	12.9%	12.3%	7.4%	7.2%	8.0%	2.0%	6.0%	10.6%	349	**P1 = 0.006**^**a**^ P2 = 0.761^a^ P3 = 0.271^a^ P4 = 0.218^a^
From a doctor	12.5%	27.3%	5.7%	5.7%	10.2%	5.7%	12.5%	11.4%	88	
From a nutritionist	9.1%	30.3%	9.1%	3.0%	15.2%	6.1%	12.1%	24.2%	33	
From a nurse	25.0%	25.0%	0.0%	0.0%	8.3%	0.0%	8.3%	8.3%	12	
Milk/yoghurt consumption										
Less than 1 cup	17.5%	14.3%	11.1%	15.9%	14.3%	4.8%	1.6%	7.9%	63	P1 = 0.197 **P2 = 0.014**^**a**^ **P3 = 0.049**^**a**^ **P4 = 0.019**^**a**^
1cup	11.2%	15.9%	7.3%	6.0%	12.1%	1.7%	9.1%	11.2%	232	
2cup	13.3%	13.3%	5.3%	1.8%	3.5%	4.4%	8.0%	8.8%	113	
3cup	12.2%	20.4%	6.1%	10.2%	4.1%	2.0%	6.1%	18.4%	49	
4cup or more	16.0%	36.0%	4.0%	0.0%	0.0%	4.0%	12.0%	24.0%	25	
Vegetables consumption										
<1 serving/day	15.2%	15.7%	8.6%	6.6%	10.2%	4.6%	9.1%	10.7%	197	P1 = 0.335^a^ P2 = 0.470^a^ P3 = 0.159^a^ P4 = 0.581^a^
1 serving/day	10.4%	17.1%	7.2%	6.8%	9.9%	1.8%	6.3%	9.9%	222	
2 serving/day	10.9%	20.0%	0.0%	5.5%	1.8%	0.0%	9.1%	21.8%	55	
3 serving/day	42.9%	0.0%	14.3%	0.0%	0.0%	14.3%	0.0%	14.3%	7	
4 serving/day	0.0%	0.0%	0.0%	0.0%	0.0%	0.0%	0.0%	0.0%	1	
Fruits consumption										
<1 serving/day	17.0%	17.0%	8.0%	10.2%	11.4%	2.3%	10.2%	12.5%	88	P1 = 0.591^a^ P2 = 0.070^a^ P3 = 0.354^a^ P4 = 0.055^a^
1 serving/day	11.7%	15.7%	9.3%	6.9%	8.9%	3.2%	5.6%	10.5%	248	
2 serving/day	14.2%	19.5%	1.8%	1.8%	8.8%	0.9%	11.5%	11.5%	113	
3 serving/day	8.0%	8.0%	8.0%	8.0%	4.0%	8.0%	0.0%	16.0%	25	
4 serving/day	0.0%	25.0%	0.0%	12.5%	0.0%	12.5%	12.5%	25.0%	8	
Protein consumption										
<1 serving/day	27.8%	16.7%	22.2%	2.8%	19.4%	2.8%	13.9%	5.6%	36	**P1 = 0.041**^**a**^ **P2 = 0.003**^**a**^ P3 = 0.126^a^ P4 = 0.080^a^
1 serving/day	11.9%	15.3%	6.8%	5.6%	8.5%	3.4%	6.2%	11.9%	177	
2 serving/day	11.8%	19.6%	5.9%	6.9%	7.8%	2.5%	6.9%	10.8%	204	
3 serving/day	11.5%	8.2%	3.3%	6.6%	8.2%	1.6%	9.8%	18.0%	61	
4 serving/day	0.0%	50.0%	0.0%	50.0%	0.0%	25.0%	25.0%	0.0%	4	
Cereals consumption										
<1 serving/day	14.8%	22.2%	25.9%	3.7%	18.5%	7.4%	7.4%	7.4%	27	P1 = 0.476^a^ **P2 = 0.019**^**a**^ P3 = 0.124^a^ P4 = 0.285^a^
1 serving/day	17.5%	19.2%	5.0%	4.2%	10.0%	2.5%	5.0%	15.0%	120	
2 serving/day	11.1%	15.7%	7.4%	8.8%	10.2%	2.8%	7.9%	9.3%	216	
3 serving/day	11.8%	14.0%	4.3%	4.3%	3.2%	2.2%	8.6%	12.9%	93	
4 serving/day	11.8%	11.8%	0.0%	5.9%	5.9%	0.0%	17.6%	23.5%	17	
5 serving/day	0.0%	0.0%	0.0%	0.0%	0.0%	0.0%	0.0%	0.0%	5	
≥6 serving/day	0.0%	50.0%	25.0%	25.0%	0.0%	25.0%	25.0%	0.0%	4	
Iodized salt consumption										
No	16.3%	17.3%	5.8%	6.7%	9.6%	1.9%	3.8%	8.7%	104	P1 = 0.645 P2 = 0.153 P3 = 0.162^a^ P4 = 0.062
Sometimes	13.0%	19.5%	3.9%	1.3%	2.6%	1.3%	6.5%	19.5%	77	
Yes	11.6%	15.6%	8.3%	7.6%	10.3%	3.7%	9.3%	10.6%	301	
Fast food consumption										
Never	17.9%	29.8%	7.5%	4.1%	11.6%	1.4%	6.8%	12.3%	146	**P1 = 0.005** P2 = 0.268 P3 = 0.228^a^ **P4 = 0.003**
Once/week	11.1%	12.3%	9.5%	6.3%	10.0%	4.2%	7.4%	8.9%	190	
2–3times/week	11.5%	14.9%	4.0%	8.7%	5.6%	2.4%	9.5%	9.5%	126	
More than3times	14.5%	10.9%	0.0%	10.0%	0.0%	5.0%	5.0%	45.0%	20	
Soft drinks consumption										
Never	14.1%	20.1%	6.7%	7.1%	11.0%	3.2%	7.1%	9.5%	283	P1 = 0.086^a^ P2 = 0.779^a^ P3 = 0.662^a^ P4 = 0.349^a^
once	11.5%	9.0%	9.8%	5.7%	7.4%	2.5%	8.2%	9.8%	122	
2_3times	9.0%	16.4%	4.5%	6.0%	4.5%	3.0%	10.4%	20.9%	67	
4_5times	25.0%	0.0%	0.0%	0.0%	0.0%	0.0%	0.0%	37.5%	8	
Daily	0.0%	50.0%	0.0%	0.0%	0.0%	0.0%	0.0%	0.0%	2	
Dietary supplements use										
No	13.1%	14.4%	7.7%	5.4%	9.2%	1.8%	8.5%	11.8%	390	**P1 = 0.024** P2 = 0.095 **P3 = 0.011** P4 = 0.479
Yes	12.0%	26.1%	4.3%	10.9%	7.6%	7.6%	4.3%	10.9%	92	
Frequency of supplement intake										
Never	13.1%	14.4%	7.7%	5.4%	9.3%	1.5%	8.5%	11.8%	390	P1 = 0.106 P2 = 0.138^a^ **P3 = 0.012**^**a**^ P4 = 0.771^a^
Irregular	13.5%	27.0%	8.1%	13.5%	7.9%	10.5%	2.7%	8.1%	37	
Regular	10.9%	25.5%	1.8%	9.1%	7.3%	7.3%	5.5%	12.7%	55	
Timing of initiation of supplement use										
Never	13.1%	14.4%	7.7%	5.4%	9.2%	1.8%	8.5%	11.8%	390	**P1 = 0.093**^**a**^ **P2 = 0.007**^**a**^ P3 = 0.055^a^ P4 = 0.237^a^
A few months ago	10.4%	26.9%	6.0%	6.0%	9.0%	7.5%	3.0%	11.9%	67	
Many months ago	16.0%	24.0%	0.0%	24.0%	4.0%	8.0%	8.0%	8.0%	25	

Each of the indices is expressed in standard deviation units (SD) from the median of the WHO Child Growth Standards adopted in 2006. Table is based on children with valid dates of birth (month and year) and valid measurement of both height and weight. Bold values indicate statistical significance.

1 Includes children who are between −2 and −3 standard deviations (SD) from the WHO Child Growth standards population median.

a Monte-Carlo corrected *p*-value, *p-value < 0.05.

P1: Height-for-age; P2: Weight-for-height; P3: Weight-for-age; P4: BMI-for-age.

Table 4 demonstrates that Children engaging in less than 30 min of outdoor play daily had the highest stunting rates (22.3% moderately stunted). Children with high screen exposure showed higher wasting rates. Finally, Screen time per day was significantly associated with stunting (*p* = 0.019) and High screen time was also significantly related to wasting (*p* = 0.041). No significant associations were found between total physical activity level and anthropometric indices (*p* > 0.05).

**Table 4:** Percentage of children under 5 years classified as malnourished according to three anthropometric indices of nutritional status: height-for-age, weight-for-height, BMI-for-Age and weight-for-age, by physical activity, Taif 2026.

Physical activity	Height for age		Weight-for-height		Weight-for-age		BMI-for-Age		Number of children (*n* = 482)	*p*-value
	Percentage below −2 SD^1^ (12.9%)	Percentage below −3 SD (16.6%)	Percentage below −2 SD (7.1%)	Percentage below −3 SD (6.4%)	Percentage below −2 SD (8.9%)	Percentage below −3 SD (2.9%)	Percentage between +2 and +3 SD (7.7%)	% above +3 SD (11.6)		
Active play time at home										
None	10.3%	15.4%	2.6%	2.6%	7.7%	0.0%	12.8%	15.4%	39	P1 = 0.707 P2 = 0.189^a^ P3 = 0.907^a^ P4 = 0.143^a^
Less than 30 min/day	11.9%	26.2%	9.5%	7.1%	11.9%	2.4%	4.8%	11.9%	42	
30 = 60 min/day	14.0%	15.8%	7.0%	3.5%	7.0%	3.5%	9.6%	15.8%	114	
1 = 2 h/day	12.6%	13.2%	9.6%	6.0%	10.2%	2.4%	8.4%	5.4%	167	
More than 2 h/day	13.3%	19.2%	4.2%	10.8%	8.3%	4.2%	4.2%	15.0%	120	
Outdoors play per day										
None	9.9%	16.5%	8.8%	4.4%	6.6%	3.3%	7.7%	13.2%	91	**P1 = 0.000** P2 = 0.920 P3 = 0.247^a^ P4 = 0.444
Less than 30 min/day	22.3%	25.6%	6.6%	6.6%	9.1%	5.8%	5.0%	14.9%	121	
30–60 min/day	9.9%	11.3%	7.9%	7.9%	12.6%	1.3%	9.9%	8.6%	151	
1–2 h/day	8.4%	13.3%	4.8%	4.8%	6.0%	1.2%	8.4%	6.0%	83	
More than 2 h/day	11.1%	16.7%	5.6%	8.3%	5.6%	2.8%	5.6%	22.2%	36	
Organized physical activity										
None	14.0%	15.8%	6.6%	6.6%	9.0%	2.1%	7.7%	11.9%	379	P1 = 0.371^a^ P2 = 0.517^a^ P3 = 0.058^a^ P4 = 0.367^a^
Less than 30 min/day	11.6%	27.9%	9.3%	11.6%	16.3%	9.3%	4.7%	11.6%	43	
30–60 min/day	9.7%	9.7%	6.5%	3.2%	0.0%	6.5%	3.2%	9.7%	31	
>1–2 h/day	4.5%	18.2%	13.6%	0.0%	9.1%	0.0%	13.6%	9.1%	22	
More than 2 h/day	0.0%	14.3%	0.0%	0.0%	0.0%	0.0%	28.6%	14.3%	7	
Screen time per day										
Meeting recommendations	13.5%	20.5%	6.5%	3.7%	8.4%	3.3%	8.4%	11.6%	215	P1 = 0.094 **P2 = 0.041** P3 = 0.862 P4 = 0.327
High Screen time	12.4%	13.5%	7.5%	8.7%	9.4%	2.6%	7.1%	11.6%	267	
Physical activity level										
<180 min/day	13.6%	17.5%	8.1%	6.9%	9.6%	3.6%	8.1%	12.0%	332	P1 = 0.367^a^ P2 = 0.290 P3 = 0.258 P4 = 0.601
≥180 min/day	11.3%	14.7%	4.7%	5.3%	7.3%	1.3%	6.7%	10.7%	150	

1 Includes children who are between −2 and −3 standard deviations (SD) from the WHO Child Growth standards population median. Bold values indicate statistical significance.

a Monte-Carlo corrected *p*-value, *p-value < 0.05.

P1: Height-for-age; P2: Weight-for-height; P3: Weight-for-age; P4: BMI-for-age.

In multivariable logistic regression analysis (Table 5), collinearity diagnostics indicated no evidence of multicollinearity among the independent variables (all VIF values were <5). The Hosmer–Lemeshow test indicated good model fit (*p* > 0.05). Formula feeding before 6 months was associated with significantly lower odds of stunting compared with exclusive breastfeeding (AOR = 0.326, 95% CI: 0.131–0.811, *p* = 0.016). Low birth weight was significantly associated with more than two-fold higher odds of stunting (AOR = 2.163, 95% CI: 1.312–3.567, *p* = 0.002). Similarly, recurrent diarrhea (AOR = 2.750, 95% CI: 1.211–6.244, *p* = 0.016) and dental caries (AOR = 2.972, 95% CI: 1.234–7.162, *p* = 0.015) were significantly increasing stunting risk, with confidence intervals excluding unity. Fast food intake was associated with significantly lower odds of stunting compared with no intake (once/week: AOR = 0.315, 95% CI: 0.139–0.712; 2–3 times/week: AOR = 0.442, 95% CI: 0.236–0.828; >3 times/week: AOR = 0.310, 95% CI: 0.122–0.788), indicating a protective association. Consuming one serving of protein per day was associated with stunting (AOR = 1.056, 95% CI: 0.090–12.330, *p* = 0.019); however, the wide confidence interval crossing 1 indicates a statistically unstable estimate, suggesting no clear or precise association compared with the reference group (>4 servings/day). Children with less than 30 min of outdoor play per day had significantly higher odds of stunting (AOR = 3.657, 95% CI: 1.761–7.595), as the confidence interval was entirely above 1.

**Table 5:** Multivariable logistic regression Models to identify the significant associated factors of children under 5 years malnutrition, Taif 2026.

Outcome	Variables	Category	B	Adjusted OR	95% CI for AOR		*p*-value
					LL	UL	
Stunting	Early feeding <6 months	Formula feeding Both breast and formula feeding	−1.120 0.058	0.326 1.060	0.131 0.546	0.811 2.056	0.016* 0.863
	Daily protein consumption	<1 serving/day 1 serving/day 2 servings/day 3 servings/day	0.054 −0.853 −0.300 −1.505	1.056 0.426 0.741 0.222	0.090 0.040 0.070 0.018	12.330 4.560 7.881 2.676	0.019* 0.481 0.804 0.236
	Low birth weight		0.772	2.163	1.312	3.567	0.002*
	Recurrent diarrhea		1.012	2.750	1.211	6.244	0.016*
	Dental caries		1.089	2.972	1.234	7.162	0.015*
	Fast food intake	Once/week	−1.155	0.315	0.139	0.712	0.006*
		2–3 times/week	−0.817	0.442	0.236	0.828	0.011*
		>3 times/week	−1.171	0.310	0.122	0.788	0.014*
	Outdoors play/day	< 30 min/day	1.297	3.657	1.761	7.595	0.001*
		30–60 min/day	0.305	1.357	0.648	2.842	0.419
		1–2 h/day	0.089	1.093	0.469	2.543	0.837
		>2 h/day	0.280	1.324	0.480	3.649	0.588
Wasting	Milk/yoghurt consumption	1 serving/day	−0.834	0.434	0.206	0.915	0.028*
		2 servings/day	−1.625	0.197	0.071	0.543	0.002*
		3 servings/day	−0.472	0.624	0.224	1.737	0.366
		>4 servings/day	−2.386	0.092	0.009	0.920	0.042*
	Daily protein consumption	1 serving/day	−2.365	0.094	0.008	1.081	0.058
		2 servings/day	−2.472	0.084	0.009	0.802	0.031*
		3 servings/day	−2.468	0.085	0.009	0.799	0.031*
		>4 servings/day	−2.573	0.076	0.007	0.815	0.033*
	High Screen time		0.635	1.887	1.012	3.520	0.046*
Underweight	Recurrent antibiotics use		−2.258	0.105	0.014	0.786	0.028*
Overweight & obesity	Recurrent diarrhea		0.871	2.390	0.061	5.384	0.035*
	Milk/yoghurt consumption	1 serving/day	1.140	3.126	1.223	7.986	0.017*
		2 servings/day	0.771	2.162	0.788	5.934	0.134
		3 servings/day	1.120	3.064	1.012	9.271	0.047*
		>4 servings/day	1.494	4.456	1.287	15.423	0.018*
	Fast food intake	Once/week	−0.142	0.868	0.475	1.587	0.646
		2–3 times/week	0.036	1.037	0.538	1.997	0.914
		>3 times/week	1.050	2.857	1.027	7.948	0.044*
	Age at introduction solid food	7–8 months	−1.204	0.300	0.119	0.759	0.011*
		9–10 months	−0.117	0.889	0.429	1.844	0.752
		After 1 year age	0.365	1.441	0.795	2.613	0.229

All relevant variables were included in the model; only statistically significant variables are presented. Model fit was assessed using the Hosmer–Lemeshow goodness-of-fit test (*p* > 0.05) # Stunting as dependent variable; X2 = 112.173; *p* < 0.001*; Nagelkerke R2 = 0.296; Significant predictors in the model: Early Feeding <6 months (Reference: Breast feeding), low birth weight (Reference: No), Recurrent diarrhea and fever (Reference: No), Dental caries (Reference: No), Daily protein consumption (Reference: > 4 servings/day), Fast food intake (Reference: No), Outdoors play per day(Reference: None). # Wasting as dependent variable; X2 = 43.710; *p* < 0.001*; Nagelkerke R2 = 0.159; Significant predictors in the model: Daily protein consumption (Reference: < 1 servings/day), daily Milk/yoghurt consumption (Reference: < 1 servings/day), High Screen time per day (Reference: Meeting recommendations) # Underweight as dependent variable; X2 = 48.98; *p* < 0.001*; Nagelkerke R2 = 0. 187; Significant predictors in the model: Recurrent antibiotics use (Reference: No). # Overweight & Obesity as dependent variable; X2 = 40.977; *p* < 0.001*; Nagelkerke R2 = 0. 130; Significant predictors in the model: Recurrent diarrhea (Reference: No), daily Milk/yoghurt consumption (Reference: < 1 servings/day), Age at introduction solid food (Reference: at 6 months), Fast food intake (Reference: No).

Higher milk/yoghurt consumption was significantly associated with reduced odds of wasting (1 serving/day: AOR = 0.434, 95% CI: 0.206–0.915; 2 servings/day: AOR = 0.197, 95% CI: 0.071–0.543; >4 servings/day: AOR = 0.092, 95% CI: 0.009–0.920). Similarly, increased daily protein intake showed a significant protective effect against wasting (2 servings/day: AOR = 0.084, 95% CI: 0.009–0.802; 3 servings/day: AOR = 0.085, 95% CI: 0.009–0.799; >4 servings/day: AOR = 0.076, 95% CI: 0.007–0.815). High screen time was associated with significantly higher odds of wasting (AOR = 1.887, 95% CI: 1.012–3.520), with the confidence interval entirely above 1. Recurrent antibiotic use was significantly associated with lower odds of underweight (AOR = 0.105, 95% CI: 0.014–0.786), as the confidence interval excluded 1.

Recurrent diarrhea was significantly associated with higher odds of overweight/obesity (AOR = 2.390, 95% CI: 0.061–5.384), although the wide confidence interval suggests limited precision in the estimate. Higher milk/yoghurt consumption was also associated with increased odds of overweight/obesity, with significant results where confidence intervals excluded 1 (1 serving/day: AOR = 3.126, 95% CI: 1.223–7.986; 3 servings/day: AOR = 3.064, 95% CI: 1.012–9.271; >4 servings/day: AOR = 4.456, 95% CI: 1.287–15.423). Fast food intake >3 times/week was associated with significantly higher odds of overweight/obesity (AOR = 2.857, 95% CI: 1.027–7.948). In contrast, introduction of solid food at 7–8 months was associated with lower odds compared with introduction at 6 months (AOR = 0.300, 95% CI: 0.119–0.759).

## 4 Discussion

The findings of this study should be interpreted within the context of the study population, as they are generalizable to children attending PHC settings rather than the broader community. It assessed the prevalence and correlates of malnutrition among children under 5 years in Taif, Saudi Arabia, using multiple anthropometric indicators including height-for-age, weight-for-height, weight-for-age, and BMI-for-age. The findings revealed that stunting affected 29.5% of children, while wasting, underweight, and obesity were observed in 13.5%, 11.8%, and 11.6%, respectively, demonstrating the coexistence of both undernutrition and overnutrition within the same population. Similar patterns have been reported globally, where rapid urbanization and dietary transitions contribute to the emergence of multiple forms of malnutrition in countries undergoing socioeconomic and nutritional transition (1). The prevalence of undernutrition observed in the present study is higher than estimates reported in earlier national studies conducted in Saudi Arabia (4, 5). A large community-based survey including more than 15,000 Saudi children reported prevalence rates of 10.9% for stunting and 9.8% for wasting, indicating that undernutrition persists among Saudi children despite improvements in healthcare services and economic conditions (4). Another national analysis highlighted substantial regional variations, with stunting reaching 13.2% in the southwestern region compared with approximately 6.4% in other regions of the Kingdom (5). More recent regional studies also demonstrate variability across Saudi Arabia. For example, a study conducted in the Jazan region in 2020 reported prevalence rates of 22% for stunting, 10.5% for wasting, and 15.9% for underweight among children under 5 years. Similarly, a study conducted in Jeddah in 2021 found wasting, stunting, and underweight among preschool children at 3.11%, 22.91%, and 20.32%, respectively (19). The prevalence observed in the current study is more comparable to findings from primary healthcare centers in the Aseer region in 2023, where wasting, underweight, stunting, and thinness were reported at 17.8%, 20.5%, 30.3%, and 18.4%, respectively (20).

Regarding overweight and obesity, national evidence from a large multicenter population-based study among children aged 2–6 years reported an obesity prevalence of approximately 12%, indicating an increasing burden of excess weight among preschool children in Saudi Arabia (6, 21, 22). The coexistence of undernutrition and overweight observed in the present study reflects the double burden of malnutrition, which has become increasingly common in countries experiencing rapid socioeconomic development. Differences between regions may be explained by variations in urbanization, socioeconomic status, dietary patterns, healthcare access, and environmental factors, all of which influence child nutritional outcomes.

When compared with regional estimates from the Middle East and North Africa (MENA) and Gulf countries, the prevalence of stunting observed in the present study appears relatively higher than reports from several high-income Gulf states but remains within the broader range reported in the MENA region. In high-income Gulf countries, stunting prevalence among children under five is generally reported between 6% and 15%, while wasting prevalence ranges from 2% to 9%, although higher levels have been documented in parts of the wider Eastern Mediterranean region (23). These differences may reflect variations in maternal education, feeding practices, socioeconomic conditions, and household environments that influence child nutritional status even within relatively high-income settings. A large geospatial analysis conducted across 73 low- and middle-income countries reported clustering of malnutrition in South Asia, the MENA region, and Sub-Saharan Africa, with prevalence estimates of 23.6% for stunting, 8.22% for wasting, and 15.5% for underweight in the MENA region (24). These findings highlight the role of socioeconomic disparities and access to health and nutrition services in shaping childhood nutritional outcomes. Many countries in the Middle East are currently undergoing a nutrition transition, characterized by increased consumption of processed foods, energy-dense diets, and reduced physical activity. This transition has contributed to the growing coexistence of undernutrition and childhood overweight, particularly in urban populations. The relatively high prevalence of overweight and obesity observed in the present study supports this emerging regional pattern.

The present study also identified several factors associated with different forms of malnutrition among children under five in Taif, highlighting the multifactorial nature of childhood nutritional status. For stunting, significant associated factors included early feeding before 6 months, low birth weight, recurrent diarrhea and fever, dental caries, inadequate protein intake, frequent fast-food consumption, and limited outdoor play. Low birth weight was strongly associated with stunting, consistent with evidence suggesting that intrauterine growth restriction and poor maternal nutrition have long-term effects on linear growth during early childhood (25). Exclusive breastfeeding was also associated with higher odds of stunting, which may reflect reverse causality, inadequate complementary feeding after 6 months, resulting in insufficient intake of energy and micronutrients despite continued breastfeeding (26, 27). Another possible explanation is insufficient monitoring of breastfeeding adequacy and child growth rather than a direct adverse effect of breastfeeding itself. Dietary and lifestyle factors also played an important role. Low daily protein intake was associated with stunting, reflecting the essential role of protein in tissue growth and immune function (28). In the present study, fast food consumption remained significantly associated with stunting even after adjustment for socioeconomic indicators and other potential confounders. This finding appears unexpected, as fast food consumption is generally linked to poorer diet quality and low micronutrient content (29). One possible explanation is the unmeasured or incompletely captured determinants of growth, such as total energy intake, meal composition, and detailed dietary quality rather than consumption food frequency assessment alone. Additionally, other unmeasured factors, parental nutritional awareness may also influence this relationship. Given the cross-sectional design, temporal ambiguity precludes any causal interpretation. Therefore, this association is more likely to represent residual confounding or underlying unmeasured dietary factors rather than a true protective biological effect of fast-food consumption on linear growth. Limited outdoor play was also associated with stunting, possibly reflecting reduced physical activity and its impact on healthy growth and musculoskeletal development (30). Additionally, recurrent diarrhea episodes were significant associated factors, as infectious diseases may impair nutrient absorption, reduce appetite, and increase metabolic demands, thereby contributing to growth faltering in early childhood (31). Poor oral health, including dental caries, may further reduce food intake due to pain during chewing.

For wasting, significant associated factors included low daily protein intake, low consumption of milk or yoghurt, and increased screen time. Wasting represents acute malnutrition resulting from recent weight loss due to inadequate dietary intake or illness. Dairy products provide high-quality protein and essential micronutrients required for growth and tissue repair, and inadequate intake has been linked with inadequate nutrient intake and increased risk of acute malnutrition (32). Excessive screen time may also influence nutritional status through sedentary behavior and unhealthy eating (33).

Interestingly, children with a history of recurrent antibiotic use showed lower odds of being underweight. This may reflect better healthcare access and timely treatment of infections, which could reduce the negative impact of illness on growth. Another possible explanation relates to antibiotic-induced alterations in gut microbiota during early childhood, which may influence nutrient metabolism and weight gain. However, the relationship between antibiotic exposure and nutritional and growth outcomes remains complex and requires further investigation (34).

Regarding overweight and obesity, some findings in the present study require cautious interpretation. Observed association between recurrent diarrhea and overweight/obesity, although seemingly counterintuitive, may reflect complex mechanisms such as alterations in gut microbiota or compensatory feeding during recovery (28, 35, 36). However, due to the cross-sectional design, causality cannot be inferred, and residual confounding may have influenced this finding. Similarly, the association between higher milk and yoghurt consumption and overweight/obesity may reflect overall dietary patterns or higher total energy intake rather than a direct effect of dairy consumption. As total caloric intake was not assessed, residual confounding cannot be excluded. Therefore, these findings should be interpreted with caution. Fast foods are typically energy-dense and high in fats and sugars, promoting weight gain during early childhood (29). Introducing complementary foods at 7–8 months was associated with lower odds of overweight and obesity compared with introduction at 6 months, suggesting that early feeding practices may influence later obesity risk through their effects on energy intake and appetite regulation (37, 38).

The findings of this study have important implications for child health policies in Taif. The observed coexistence of undernutrition and overweight highlights the need for an integrated child nutrition strategy addressing both forms of malnutrition. At the primary healthcare level, routine growth monitoring should be strengthened, with early identification of high-risk children, particularly those with low birth weight and recurrent infections. Nutritional counseling should be integrated into antenatal and postnatal care to improve infant feeding practices and dietary diversity. Targeted interventions are also needed to improve dietary habits and promote adequate protein intake, alongside school and community-based health education addressing unhealthy dietary habits, excessive screen time, and low physical activity. In addition, integrating nutrition services with infection prevention and oral health programs is essential within primary healthcare settings. Finally, a multisectoral approach involving health, education, and municipal authorities is required to promote healthier food environments and improve child nutrition outcomes in Taif.

## 5 Strengths and limitations

This study has several strengths. First, it provides updated evidence on the burden and determinants of multiple forms of malnutrition among children under 6 years in Taif, an area where limited recent data are available. Second, the study assessed malnutrition using multiple standardized anthropometric indicators (height-for-age, weight-for-height, weight-for-age, and BMI-for-age), allowing a comprehensive evaluation of both undernutrition and overweight. Third, a wide range of potential determinants including sociodemographic characteristics, feeding practices, dietary habits, health, and lifestyle behaviors were examined, enabling identification of modifiable associated factors that may inform targeted public health interventions. However, several limitations should be considered when interpreting the findings. The cross-sectional design limits the ability to establish causal relationships between the identified associated factors and nutritional outcomes, and the temporal relationship between variables cannot be determined, raising the possibility of reverse causation. The study was conducted among children attending PHCs; therefore, the findings are generalizable only to this population, which may introduce selection bias if compared to community-based samples. Although PHCs were randomly selected, the inclusion of only four centers may limit the representativeness of the sample at the city level. Some variables, including feeding practices and illness history, were based on caregiver self-report, which may be subject to recall and social desirability bias, which is an inherent limitation of retrospective data collection. In addition, assessment of dietary habits using frequency questionnaire only, may not reflect actual intake and overall dietary quality. Although several relevant factors were included in the multivariable models, residual confounding cannot be excluded. Given the exploratory cross-sectional design of this study, no adjustment for multiple comparisons was applied, which may increase the likelihood of type I error. Moreover, although all regression assumptions were assessed and model diagnostics indicated acceptable fit, the possibility of residual overfitting due to multiple predictors cannot be fully excluded. Finally, future studies with larger sample sizes are warranted to explore potential heterogeneity across subgroups and confirm the robustness of the observed associations. Despite these limitations, the study provides valuable insights into the magnitude and complex determinants of childhood malnutrition and highlights important areas for future research and intervention.

## 6 Conclusion

This study highlights that malnutrition among children under 5 years in Taif remains a significant public health concern, with the coexistence of undernutrition and overweight reflecting a double burden of malnutrition. The relatively high prevalence of stunting, along with notable levels of wasting, underweight, and obesity, indicates that both traditional and emerging determinants influence child nutritional status in this population. Several modifiable factors were identified, including low birth weight, recurrent childhood infections, suboptimal infant feeding practices, inadequate dietary protein intake, and unhealthy lifestyle behaviors including high screen time and low physical activity. These results highlight the importance of implementing context-specific, primary health care–based strategies aimed at improving infant feeding practices, dietary habits, lifestyle behaviors, and enhancing the prevention and appropriate management of recurrent childhood illnesses, with rational use of antibiotics. Strengthening and optimizing existing maternal and child health services, including antenatal care, routine growth monitoring, and caregiver-focused nutrition counseling within primary health care settings, may improve the early detection and management of both undernutrition and overnutrition in early childhood.

## Data Availability

The datasets generated and analyzed during the current study are not publicly available but are available from the corresponding author upon reasonable request. Requests to access the datasets should be directed to NE-F, nesrin_kamal@yahoo.com.
